# Reduced mechanical efficiency in left‐ventricular trabeculae of the spontaneously hypertensive rat

**DOI:** 10.14814/phy2.12211

**Published:** 2014-11-20

**Authors:** June‐Chiew Han, Kenneth Tran, Callum M. Johnston, Poul M. F. Nielsen, Carolyn J. Barrett, Andrew J. Taberner, Denis S. Loiselle

**Affiliations:** 1 Auckland Bioengineering Institute, The University of Auckland, Auckland, New Zealand; 2 Department of Engineering Science, The University of Auckland, Auckland, New Zealand; 3 Department of Physiology, The University of Auckland, Auckland, New Zealand

**Keywords:** Cardiac efficiency, cardiac hypertrophy, heart failure, hypertension

## Abstract

Long‐term systemic arterial hypertension, and its associated compensatory response of left‐ventricular hypertrophy, is fatal. This disease leads to cardiac failure and culminates in death. The spontaneously hypertensive rat (SHR) is an excellent animal model for studying this pathology, suffering from ventricular failure beginning at about 18 months of age. In this study, we isolated left‐ventricular trabeculae from SHR‐F hearts and contrasted their mechanoenergetic performance with those from nonfailing SHR (SHR‐NF) and normotensive Wistar rats. Our results show that, whereas the performance of the SHR‐F differed little from that of the SHR‐NF, both SHR groups performed less stress‐length work than that of Wistar trabeculae. Their lower work output arose from reduced ability to produce sufficient force and shortening. Neither their heat production nor their enthalpy output (the sum of work and heat), particularly the energy cost of Ca^2+^ cycling, differed from that of the Wistar controls. Consequently, mechanical efficiency (the ratio of work to change of enthalpy) of both SHR groups was lower than that of the Wistar trabeculae. Our data suggest that in hypertension‐induced left‐ventricular hypertrophy, the mechanical performance of the tissue is compromised such that myocardial efficiency is reduced.

## Abstract (precis)

Our study provides the first comprehensive examination of cardiac mechanoenergetics in the spontaneously hypertensive rat (SHR – a widely utilized model of human hypertensive cardiomyopathy). Simultaneous measurement of force development, muscle shortening and heat production allows us to calculate force‐length work output, change in enthalpy and, ultimately, mechanical efficiency. In comparison to their age‐matched normotensive Wistar controls, trabeculae from SHR animals, whether “nonfailing” or “failing”, show reduced ability to perform work, despite only modest reduction in heat production. In consequence, their efficiency deteriorates.

## Introduction

Systemic hypertension is known to be associated with pathologic left‐ventricular hypertrophy. Long‐term effects of this disease are fatal as they lead to cardiac failure, ultimately culminating in death. In patients with hypertensive‐hypertrophy, reduced myocardial efficiency at the whole‐organ level has consistently been reported (Ishibashi et al. 1996; Laine et al. 1999; Akinboboye et al. 2004; de las Fuentes et al. 2006). However, the underlying mechanoenergetic basis leading to hypertensive‐hypertrophic failure remains uncertain. To gain insight into the cause of mechanoenergetic failure, it is advantageous to use isolated ventricular tissues. Tissue models can be subjected to a wide range of work‐loads, achieved by changing their lengths or by varying afterloads, neither of which is possible when studying the human heart in vivo.

We chose to use the spontaneously hypertensive rat (SHR). It is a laboratory model of genetic systemic hypertension that mimics human essential hypertension (Trippodo and Frohlich 1981; Doggrell and Brown 1998). Prolonged periods of hypertension and the attendant development of left‐ventricular hypertrophy in the SHR ultimately lead to ventricular failure, beginning at about 18 months of age. Early physical signs of heart failure in failing SHR (“SHR‐F”) include tachypnea and labored respiration. On pathological examination, SHR‐F is found to have pleural effusion and left atrial thrombi. Previous experimental studies have revealed numerous pathophysiologic changes in SHR‐F myocardium. Ionic and electrical changes include reduced transient outward K^+^ current (Cerbai et al. 1994), prolonged action potential duration (Cerbai et al. 1994), prolonged Ca^2+^ transient duration (Bing et al. 1991; Ward et al. 2003, 2010; Wasserstrom et al. 2009; Kapur et al. 2010), and reduced expression of SERCA and Na^+^‐K^+^ exchanger (Ward et al. 2010). Biochemical changes include increase in the “slow” isoform of myosin heavy chain (Boluyt et al. 1994) and myosin isozymes (Bing et al. 1991). Histological studies show T‐tubule disruption (Ward et al. 2010; Shah et al. 2014), increased myocardial fibrosis (Conrad et al. 1995; Shah et al. 2014), disordered laminar arrangement of myocytes (LeGrice et al. 2012), and increased apoptosis of cardiomyocytes (Li et al. 1997) and noncardiomyocytes (Ikeda et al. 1999). Alteration in gene expression has also been reported (Brooks et al. 2010). These microscopic changes are consistent with the macroscopic myocardial functional changes in the SHR‐F preparations: (1) mechanical dysfunction, as indexed by both impaired contractility (Bing et al. 1991, 1995; Conrad et al. 1991, 1995; Brooks et al. 1993, 2010; Ward et al. 2003, 2010) and increased passive stiffness (Bing et al. 1995; Conrad et al. 1995; Brooks et al. 2010); and (2) energetic compromise, as indexed by reduced oxygen consumption (Brooks et al. 1993).

Despite extensive studies of the SHR‐F, it is uncertain whether it has reduced mechanical efficiency. Thus, in this study, we determined, for the first time, mechanical efficiency of the SHR‐F. We compared their mechanoenergetic performance with that of nonfailing SHR (“SHR‐NF”) and normotensive Wistar control trabeculae, in order to probe the mechanoenergetic effect of hypertensive‐hypertrophy.

## Methods

Experiments were conducted in accordance with protocols approved by The University of Auckland Animal Ethics Committee. Under the terms of this Approval, an animal showing signs of cardiovascular or respiratory distress must be euthanized as soon as possible – whether or not its tissues can be salvaged for experimentation. It is for this reason that animals in the “SHR‐F” Group (see below) were included in the study in the absence of accompanying measurements of blood pressure.

### Animal

Upon reaching 18 months of age, male spontaneously hypertensive rats (SHR) were studied (within 2 days) if signs of heart failure (progressive loss of body mass, tachypnea, and labored breathing) were noted by Animal Welfare personnel. During the excision and cannulation of the heart, heart failure was further confirmed by the presence of pleural effusion and left atrial thrombi. Animals in this failing SHR group, with such clinical and pathological symptoms, were labeled “SHR‐F”, consistent with previous studies (Bing et al. 1991, 1995; Conrad et al. 1991, 1995). A group of nonfailing SHR (labeled “SHR‐NF”) and their age‐matched Wistar rats (labeled “Wistar”) completed this three‐group study.

### Measurement of blood pressure

Rats from the Wistar (*n* = 4) and SHR‐NF (*n* = 3) groups were randomly selected for in vivo telemetric measurement of blood pressure, the details of which have been previously described (Han et al. 2014a). Rats were monitored for 2 weeks postsurgery; data from the second week are presented. For ethical reasons, no blood pressure measurements were taken from the SHR‐F group because they had to be used within 2 days upon noticing symptoms of heart failure. Previous studies (Conrad et al. 1991; Bing et al. 1995), using the tail‐cuff method, showed that the blood pressure of the SHR‐F stayed high, comparable to that of the SHR‐NF.

### Preparation of trabeculae

As described previously (Goo et al. 2014; Han et al. 2014b), each rat was deeply anesthetized with isoflurane (5% in O_2_), and its body mass measured prior to injection with heparin (1000 IU·kg^−1^). Following cervical dislocation, the excised heart was plunged into chilled Tyrode solution, and the aorta immediately cannulated for Langendorff perfusion with Tyrode solution (in mmol·L^−1^: 130 NaCl, 6 KCl, 1 MgCl_2_, 0.5 NaH_2_PO_4_, 1.5 CaCl_2_, 10 HEPES, 10 glucose; pH adjusted to 7.4 by addition of Tris) vigorously gassed with 100% O_2_ at room temperature. After examining whole‐heart mechanoenergetics, using techniques previously described (Han et al. 2014a), the heart was Langendorff‐perfused with dissection solution (Tyrode solution with Ca^2+^ reduced to 0.3 mmol·L^−1^ and supplemented with 20 mmol·L^−1^ 2,3‐butanedione monoxime). Trabeculae were isolated from the inner wall of the left ventricle (LV) and mounted in our work‐loop calorimeter (Taberner et al. 2011). While the trabecula was being superfused (at a rate of 0.5 *μ*L·sec^−1^–0.7 *μ*L·sec^−1^) with Tyrode solution at 32°C and electrically stimulated to contract at 3 Hz, it was gradually stretched to optimal length (*L*_o_; the length that maximizes developed force). At *L*_o_, the length and diameter of the trabecula were then measured using a microscope graticule. In total, 18 Wistar, 14 SHR‐NF and 7 SHR‐F trabeculae (isolated from 16 Wistar, 12 SHR‐NF, and 5 SHR‐F hearts, respectively) were examined.

### Experimental protocol

Each experiment commenced with a trabecula contracting isometrically at *L*_o_ while being paced (typically 3 V–3 msec stimulus pulses) at 3 Hz, with the calorimeter system maintained at a temperature of 32°C. We chose this combination of experimental conditions (3 Hz at 32°C) in order to avoid incomplete relaxation of the twitch.

Each trabecula was first required to undergo work‐loop contractions at preload *L*_o_ and at eight different afterloads (presented in decreasing order; the maximal afterload was equivalent to the isometric developed force, whereas the minimal afterload was in the vicinity of zero developed force). The work‐loop protocol was designed to approximate the auxotonic pressure‐volume work‐loop of the heart. Steady‐state (2–3 min upon a change of experimental interventions) values of force‐length work output (calculated as the area within the work‐loop), work‐loop heat output, extent of shortening, velocity of shortening, power of shortening, and mechanical efficiency (the ratio of work to the sum of work and heat) were the principal variables of interest. Upon completion of the work‐loop protocol, each trabecula was then subjected to isometric contractions at seven different preloads at progressively diminishing muscle lengths, commencing at *L*_*o*_ and proceeding to a slack length which produced essentially zero developed force. Steady‐state isometric force, twitch duration, maximal rates of rise and fall of the twitch, force‐time integral (the area under the twitch), and isometric heat output were quantified. Muscle force was converted to stress by dividing by muscle cross‐sectional area (calculated from a single‐view measurement of muscle diameter and assumed circular cross‐section).

### Corrections for thermal artefacts

Upon completion of the two distinct protocols, as described above, stimulation was discontinued and the trabecula remained quiescent. The heat artefact arising from the cyclical movement of the upstream hook (required to change the length of the trabecula in order to perform a series of work‐loop contractions) was determined – typically <10% of the peak total measured signal (observed when the trabecula developed peak isometric stress). The trabecula was then removed from the calorimeter and the heat artefact resulting from electrical stimulation was measured – typically about 10% (quantified at 3 Hz, 3 V and 3 msec stimulus pulses) of the peak total measured signal. Net muscle heat output was corrected for these two sources of heat artefact.

### Statistical analyses

Data were fitted using either first‐, second‐, or third‐order (some constrained to pass through the zero intercept) polynomial regression. A lower order polynomial regression (i.e., linear) was used if an *F*‐test detected no statistical improvement of the fit vis‐à‐vis a higher order polynomial (i.e., quadratic). The regression lines (each fitted to the data obtained from a single trabecula) were averaged within groups using the “random coefficient model” within *Proc Mixed* of the SAS package, which assumes that the regression coefficients arise from a multivariate normal probability distribution (Feldman 1988; Littell et al. 2006). The underlying statistics for this regression‐averaging approach have been detailed elsewhere (Feldman 1988). Differences among regression lines of the Wistar, SHR‐NF, and SHR‐F groups were examined for statistical significance using a set of orthogonal contrast vectors, that is, [2 −1 −1] and [0 1 −1]. On each plot, significant statistical effects (*P* < 0.05) labeled “Strain” and “Failing” denote the two orthogonal contrast vectors, respectively. The mean ± standard error (SE) of the peak value of a parameter (where the greatest SE occurs) was also superimposed on each plot.

## Results

### Blood pressures in vivo

Compared with the Wistar rats, the SHR‐NF had greater average values of systolic, diastolic, and mean arterial blood pressures (Table 1).

**Table 1.** Blood pressure in vivo of the nonfailing spontaneously hypertensive rat (SHR‐NF) and Wistar rats.

Blood pressure	Wistar (*n* = 4)		SHR‐NF (*n* = 3)	
	kPa	mmHg	kPa	mmHg
Systolic	19.45 ± 1.21	145.9 ± 9.1	25.09 ± 0.55*	188.2 ± 4.1*
Diastolic	12.73 ± 1.29	95.5 ± 9.7	16.81 ± 0.27*	126.1 ± 2.0*
Mean arterial	15.72 ± 1.24	117.9 ± 9.3	20.56 ± 0.28*	154.2 ± 2.1*

Values are mean ± SE.

* *P* < 0.05, Wistar versus SHR‐NF.

### Rat model

As reported in Table 2, the SHR‐F were, on average, 68 days older than both the SHR‐NF and Wistar rats. The age of the SHR‐F ranged from 533 days to 652 days. Thus, we chose to study the SHR‐NF (and correspondingly the age‐matched Wistar controls) when they reached 540–560 days, before they developed heart failure. Our study thus investigated the transition from compensated left‐ventricular hypertrophy to heart failure in the SHR.

**Table 2.** General characteristics of the nonfailing spontaneously hypertensive rat (SHR‐NF), failing SHR (SHR‐F) and Wistar rats.

Parameter	Wistar (*n* = 16)	SHR‐NF (*n* = 12)	SHR‐F (*n* = 5)	Significant statistical effect
Age (day)	549 ± 2	549 ± 2	617 ± 22	Strain, Failing
Body mass (g)	760 ± 37	463 ± 9	380 ± 24	Strain
Tibial length (mm)	48.5 ± 0.5	45.3 ± 0.4	41.6 ± 0.7	Strain, Failing
Lung mass (g)	2.51 ± 0.25	2.28 ± 0.16	2.52 ± 0.28	–
Lung mass/body mass (%)	0.36 ± 0.06	0.49 ± 0.03	0.68 ± 0.11	Strain
Heart mass (g)	1.99 ± 0.10	2.10 ± 0.04	2.35 ± 0.18	–
Heart mass/body mass (%)	0.27 ± 0.02	0.45 ± 0.01	0.63 ± 0.07	Strain, Failing
RV wall thickness (mm)	1.36 ± 0.06	0.93 ± 0.04	1.24 ± 0.16	Strain, Failing
Septal wall thickness (mm)	3.73 ± 0.09	4.07 ± 0.08	3.76 ± 0.19	–
LV wall thickness (mm)	4.14 ± 0.07	4.22 ± 0.08	4.12 ± 0.08	–
RV thickness/tibial length (%)	2.82 ± 0.13	2.07 ± 0.09	2.99 ± 0.40	Failing
Septal thickness/tibial length (%)	7.69 ± 0.20	8.98 ± 0.18	9.05 ± 0.52	Strain
LV thickness/tibial length (%)	8.54 ± 0.12	9.31 ± 0.16	9.93 ± 0.35	Strain, Failing

Values are mean ± SE.

Effect of “Strain”: Wistar versus average of both SHR groups.

Effect of “Failing”: SHR‐F versus SHR‐NF.

### Morphometric characteristics of the rats

As documented in Table 2, compared with Wistars, both SHR‐NF and SHR were smaller, as indicated by their lower average body masses and shorter average tibial lengths. The SHR‐F had smaller average tibial length than that of the SHR‐NF. When normalized for body mass, the hearts, and lungs of the SHR were larger than those of the Wistar rats. Whereas the average lung mass per body mass of the SHR‐F was not different from that of the SHR‐NF (*P* = 0.07), the average heart mass per body mass of the SHR‐F was greater than that of the SHR‐NF. When normalized for tibial length, the RV wall of the SHR‐F was thicker than that of the SHR‐NF. Both SHR‐NF and SHR‐F clearly demonstrated LV hypertrophy, as evidenced by their thicker LV wall per tibial length. The SHR‐F demonstrated even greater LV hypertrophy than the SHR‐NF.

### Dimensions of the experimented trabeculae

The average diameter and the average length of Wistar trabeculae were greater than those of SHR‐NF trabeculae and SHR‐F trabeculae (Table 3).

**Table 3.** Dimensions of Wistar, nonfailing spontaneously hypertensive rat (SHR‐NF) and failing SHR (SHR‐F) trabeculae.

Parameter	Wistar (*n* = 18)	SHR‐NF (*n* = 14)	SHR‐F (*n* = 7)	Significant statistical effect
Diameter (*μ*m)	317 ± 18	284 ± 14	264 ± 18	Strain
Length (mm)	3.58 ± 0.17	2.50 ± 0.16	2.30 ± 0.15	Strain

Values are mean ± SE.

Effect of “Strain”: Wistar versus average of both SHR groups.

Effect of “Failing”: SHR‐F versus SHR‐NF.

### Isometric contractions

**Figure 1.**
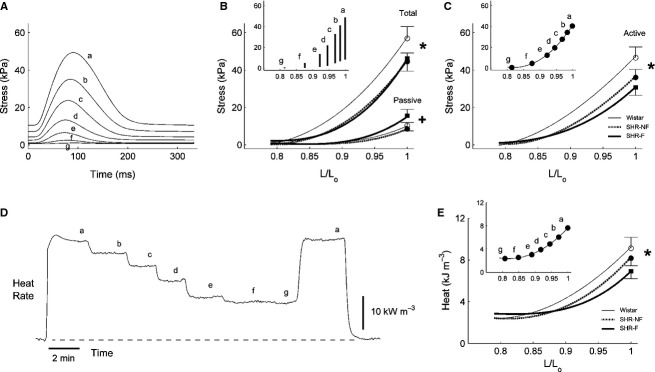
Isometric total and active stress‐length relations and heat‐length relation. (A) Records from a representative failing spontaneously hypertensive rat (SHR‐F) trabecula of steady‐state isometric twitches as functions of decreasing muscle length (a–g, where “a” represents the isometric developed stress at *L*_o_). (B) Average total and passive stresses as functions of relative muscle length (*L*/*L*_o_) obtained by fitting cubic regressions, respectively, to the peak stress and passive stress data; the inset plots stress development as a function of *L*/*L*_o_ of a representative trabecula. (C) Average active (total minus passive) stress‐length relations; the inset shows data from the same trabecula. (D) Record of rate of heat output from a representative trabecula subjected to variable muscle‐length isometric contractions. (E) Heat per twitch (rate of heat production divided by stimulus frequency) as function of *L*/*L*_o_. The symbol “*” denotes significant effect of “Strain” (i.e., comparing the mean regression line of the Wistar group with the average of the regression lines of both the nonfailing SHR (SHR‐NF) and SHR‐F groups); “+” denotes significant effect of “Failing” (i.e., comparing the mean regression line of the SHR‐F group with that of the SHR‐NF group). Data (mean ± SE) at *L*_o_ were superimposed on appropriate panels to demonstrate the variability of each average regression line.

**Figure 2.**
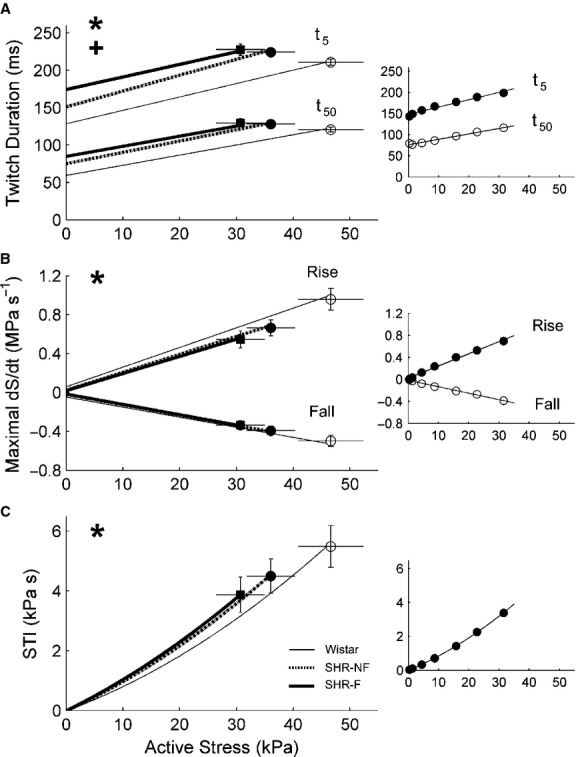
Kinetics of isometric contractions at steady states. (A) Twitch durations at 5% (*t*_5_) and at 50% (*t*_50_) of peak stress as functions of active stress. (B) Maximal rate of rise (+dS/dt) and rate of fall (−dS/dt) of active stress, computed, respectively, from the ascending and descending limbs of the twitch, were plotted as functions of active stress. (C) Stress‐time integral (STI; the area under the twitch) as a function of active stress. *P < 0.05 for Wistar versus both spontaneously hypertensive rat (SHR) groups, ^+^*P* < 0.05 for failing SHR (SHR‐F) versus nonfailing SHR (SHR‐NF). Data (mean ± SE) at *L*_o_ were superimposed on appropriate panels to demonstrate variability of each average regression line. The insets show data from a representative SHR‐NF trabecula.

**Figure 3.**
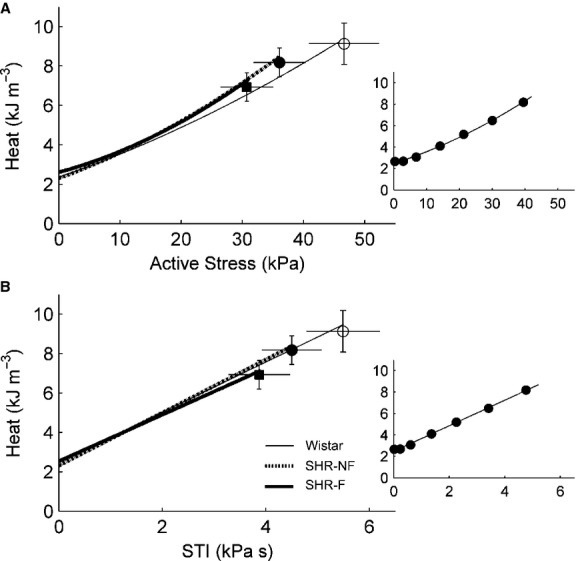
Steady‐state isometric heat‐stress and heat‐STI relations. (A) Average relations between heat per twitch and active stress. Isometric activation heat was predicted from the y‐intercept of the isometric heat‐stress relation. (B) Average relations between heat per twitch and stress‐time integral (STI). There were no statistical differences in these relations among the rat groups. Data (mean ± SE) at *L*_o_ were superimposed on appropriate panels to demonstrate variability of each average regression line. The insets show data from a representative failing spontaneously hypertensive rat (SHR‐F) trabecula.

Both groups of SHR trabeculae produced lower stresses than that of Wistar trabeculae, as evidenced by their lower average total and average active stress‐length relations (Fig. 1). The average passive stress‐length relation of the SHR‐F was steeper than the SHR‐NF groups (Fig. 1B). The average heat‐length relations of both SHR groups were also lower than the Wistar group (Fig. 1E). Compared with the Wistar group, both SHR groups also demonstrated prolonged twitch duration (quantified at 5% and at 50% of peak stress), had lower maximal rates of rise and fall of twitch stress, and had smaller values of stress‐time integral (STI, the area under the twitch), as indicated by the respective relations shown in Figure 2. Given these mechanical differences, the heat production (plotted as functions of active stress and STI) of the SHR trabeculae was, surprisingly, not different from that of the Wistar trabeculae (Fig. 3). The average activation heat (predicted by the y‐intercept of the heat‐stress relation; Fig. 3A) was not different among the rat groups. The average values of activation heat were 2.35 kJ·m^−3^± 0.27 kJ·m^−3^, 2.30 kJ·m^−3^ ± 0.30 kJ·m^−3^, and 2.61 kJ·m^−3^ ± 0.43 kJ·m^−3^, respectively for the Wistar, SHR and SHR‐F groups. These values were not different from those predicted from the heat‐STI relation (Fig. 3B; respectively 2.49 kJ·m^−3^ ± 0.27 kJ·m^−3^, 2.31 kJ·m^−3^ ±0.31 kJ·m^−3^, and 2.54 kJ·m^−3^ ± 0.44 kJ·m^−3^).

### Work‐loop contractions

**Figure 4.**
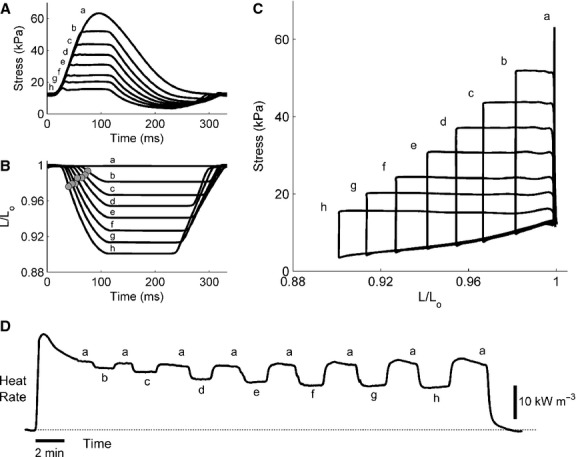
Raw records of work‐loop contractions from representative trabeculae. (A) Isometric twitch (a) superimposed with seven work‐loop twitches of various afterloads (b–h). (B) Corresponding length change throughout the time‐course of twitches in *A*. Gray circles indicate locations at which velocities of muscle shortening were maximal. (C) Parametric plots of the data in *A* against those in *B*. The area within the stress‐length loop quantifies work output, as calculated by integrating stress as a function of *L*/*L*_o_ over the entire period of the twitch. Note that for the isometric contraction (“a”), work output is zero. (D) Rate of heat production for seven variously‐afterloaded work‐loop contractions (b–h), bracketed by eight isometric contractions (a).

**Figure 5.**
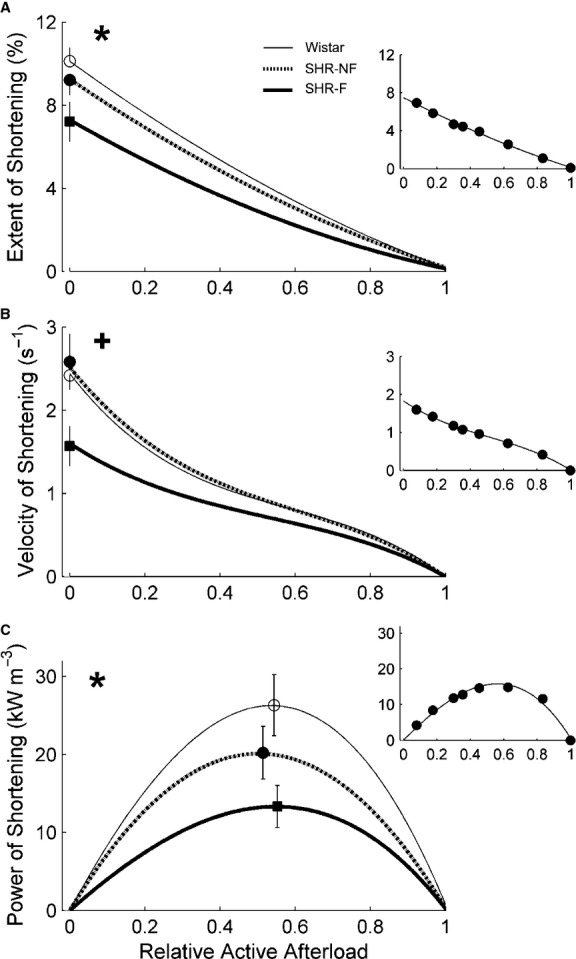
Shortening kinetics obtained from work‐loop contractions. (A) Maximal extent of shortening (calculated from the end‐systolic length in Fig. 4C), (B) maximal velocity of shortening (at the times indicated by the circles in Fig. 4B), and (C) maximal power of shortening (calculated as the product of maximal velocity of shortening and active afterload, where active afterload is afterload minus peak passive stress), as functions of relative active afterload. *P < 0.05 for Wistar versus both spontaneously hypertensive rat (SHR) groups, ^+^*P* < 0.05 for failing SHR (SHR‐F) versus nonfailing SHR (SHR‐NF). Peak values (mean ± SE) of variables were superimposed on appropriate panels as an indication of the variability of each average regression line. Note that the greatest SE occurred at peak values. The insets show data from a representative SHR‐F trabecula.

**Figure 6.**
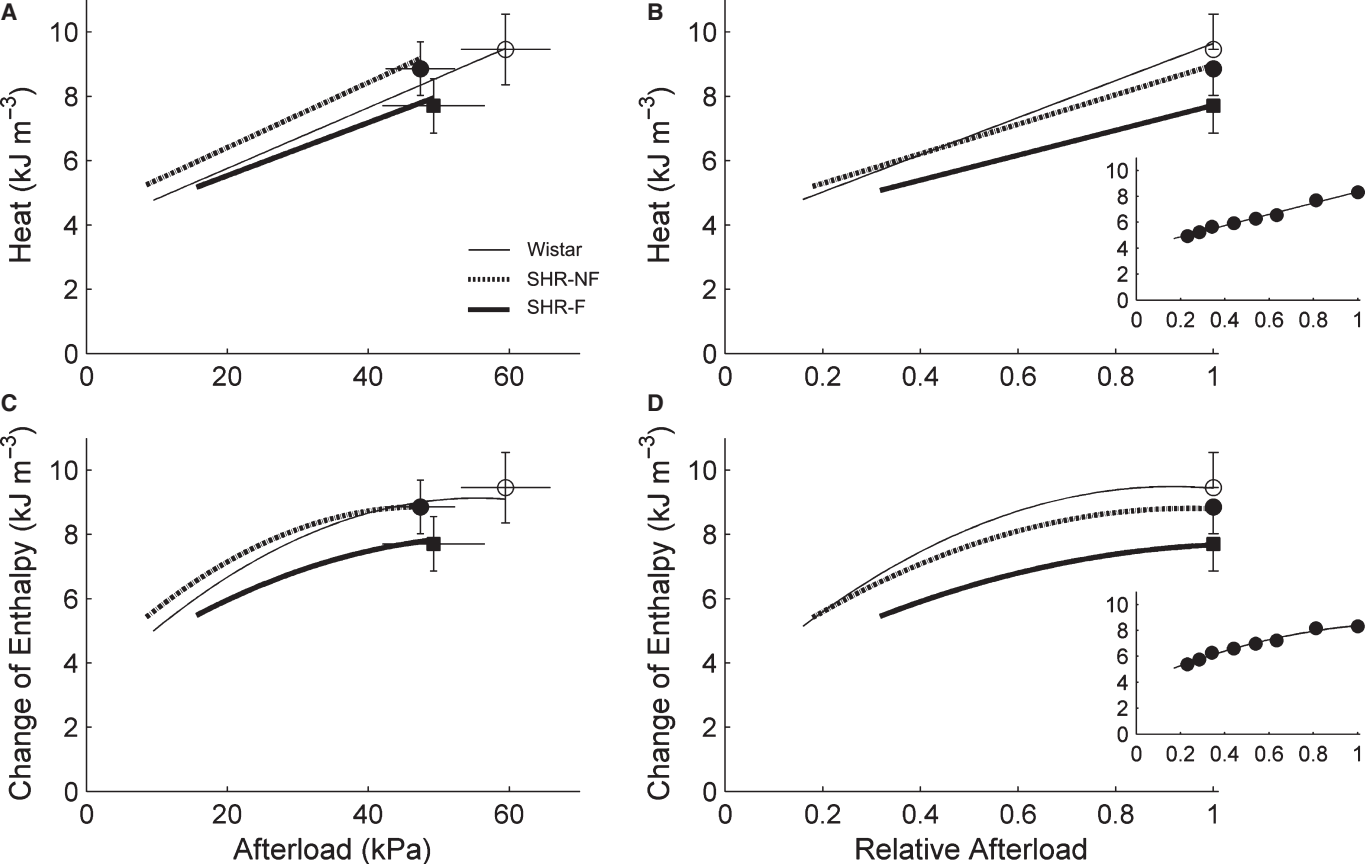
Heat production and change of enthalpy of work‐loop contractions. Average relations between heat production or change of enthalpy as functions of afterload (A and C, respectively) or relative afterload (B and D, respectively). There were no significant differences between the rat groups. Peak values (mean ± SE) of variables were superimposed on appropriate panels as an indication of the variability of each average regression line. Note that the greatest SE occurred at peak values. The insets show data from a representative failing spontaneously hypertensive rat (SHR‐F) trabecula.

**Figure 7.**
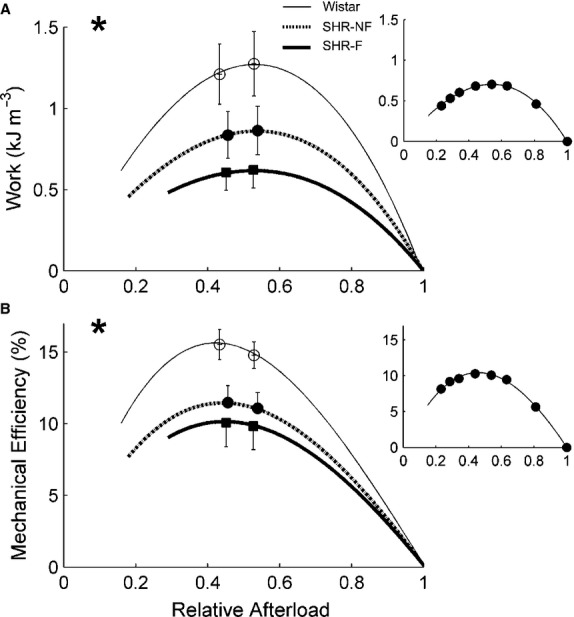
Work output and efficiency as functions of relative afterload. Average dependences of work (A) and mechanical efficiency (B) on relative afterload. *P < 0.05 for Wistar versus both spontaneously hypertensive rat (SHR) groups, ^+^*P* < 0.05 for failing SHR (SHR‐F) versus nonfailing SHR (SHR‐NF). Note that peak mechanical efficiency occurred at relative afterloads (0.45) lower than that for peak work (0.52). Peak values (mean ± SE) of variables were superimposed on appropriate panels as an indication of the variability of each average regression line. Note that the greatest SE occurred at peak values. The insets show data from a representative SHR‐F trabecula.

Work‐loop contractions (Fig. 4), simplified versions of the contraction patterns of the heart, allow quantification of shortening‐related parameters (Fig. 5) and stress‐length work output (Figs. 4C, 7A). Coupled with simultaneous measurement of heat production (Figs. 4D, 6), work‐loop contractions allow quantification of enthalpy output (the sum of work and heat; Fig. 6) and hence mechanical efficiency (the ratio of work to enthalpy output; Fig. 7B and C).

In Figure 5A, the relation between extent of shortening and relative active afterload was lower in the SHR compared with that of the Wistar trabeculae. However, that for the SHR‐F did not differ significantly from that of the SHR‐NF (*P* = 0.16). Similar results were obtained for the y‐intercept (i.e., the peak value of extent of shortening; *P* = 0.10). For comparison of velocity of shortening among groups (calculated from the length‐time trace during the shortening phase of the work‐loop, Fig. 4B), its relation with relative active afterload and its peak value were both lower for the SHR‐F group (Fig. 5B). Its product with active afterload, which yielded the power of shortening (Fig. 5C), was lower for the SHR trabeculae (when considering either its relation with relative active afterload or its peak value); this relation for the SHR‐F trabeculae did not differ from that of the SHR‐NF (*P* = 0.12).

There were no differences among rat groups in heat production or enthalpy output as functions of either afterload or relative afterload (Fig. 6). However, there were differences among rat groups in work (*W*) output and mechanical efficiency. These two indices (peak values, as well as functions of relative afterload) were lower for the two SHR groups than those for the Wistar group (Fig. 7). Peak values of mechanical efficiency were 15.5% ± 1.1%, 11.5% ± 1.2%, and 10.1% ± 1.7% for the Wistar, SHR‐NF, and SHR‐F groups, respectively.

## Discussion

This study examines the mechanoenergetic performance of the spontaneously hypertensive rat (SHR) at end‐stage hypertrophy‐induced left‐ventricular failure (SHR‐F). Whereas previous studies compared muscle performance at single values of afterload (commonly at optimal muscle length), we compared values among rat groups across a range of afterloads.

Compared with Wistar controls, SHR trabeculae demonstrate prolongation of twitch duration (Fig. 2A) and lower rates of rise and fall of the isometric twitch (Fig. 2B). These results are consistent with previously reported ionic and electrical impairments (see Introduction). Failing SHR (SHR‐F) trabeculae develop lower stress, have higher passive stress (Fig. 1B), and shorten less (Fig. 5A), consistent with the histological results described in the Introduction. Furthermore, the SHR‐F trabeculae shorten with lower velocity (Fig. 5B), again consistent with biochemical data outlined in the Introduction.

Since the SHR‐F trabeculae have reduced ability to produce stress and to shorten, their work output is lower (Fig. 7A). However, their heat production, particularly the energy cost of cycling of Ca^2+^ by the sarcoplasmic reticulum Ca^2+^‐ATPase (SERCA) and to a lesser extent the energy cost of Na^+^ removal by the sarcolemmal Na^+^‐K^+^‐ATPase (estimated from the y‐intercept of the isometric heat‐stress relation; Fig. 3A), are normal (Fig. 6). Given their reduced work output and essentially normal enthalpy output (the sum of heat and work), mechanical efficiency (the ratio of work to enthalpy output) of the SHR‐F trabeculae is lower (Fig. 7B). In summary, our results show that the SHR‐F trabeculae, despite normal energetics of SERCA and the Na^+^‐K^+^‐ATPase, have reduced ability to produce sufficient force and to undergo sufficient shortening, which gives rise to reduced mechanical efficiency. Our results further suggest that the nonfailing SHR (SHR‐NF) trabeculae are in transition to failure, given little difference in mechanoenergetic performance vis‐à‐vis the SHR‐F trabeculae.

### Reduced mechanical efficiency in the SHR

Since we are the first to have *explicitly* quantified the mechanical efficiency of the SHR, we opt to compare our results with those studies that *implicitly* provided data on the efficiency of the SHR. Our results (lower work, normal change of enthalpy, and hence lower efficiency), obtained from aged rats (older than 18 months), are not consistent with those *hinted* by three published studies, which compared the performance of SHR with Wistar‐Kyoto rats at their adult stage (6–12 month old). These three studies (Friberg and Nordlander 1986; Tubau et al. 1987; Watters et al. 1989) found lower change of enthalpy (indexed by oxygen consumption) with either greater or unchanged “work output” by the adult SHR hearts, which *implicitly* suggests that mechanical efficiency is greater in the adult SHR.

Our results of normal enthalpy output in the aged SHR are in agreement with those of Burns and Montini (1982) who reported no difference in oxygen consumption between the hearts of the SHR and Wistar‐Kyoto rats of young age (4‐month old). However, their data show no difference in either the slopes or the intercepts of the relations describing oxygen consumption and “work output”, which may suggest that myocardial efficiency is unchanged in young SHR.

It can be argued that the discrepant findings between our results (from aged SHR) and those studies of young and adult SHR may arise from our use of Wistar rats as the generic control for SHR. But, it has been demonstrated by Bing et al. (1988) that there is no difference in mechanical performance between LV papillary muscles isolated from the Wistar and Wistar‐Kyoto hearts. More importantly, we decided against using the Wistar‐Kyoto rats as controls because, unlike Wistar rats, they have been shown to develop hypertension‐independent LV hypertrophy (Aiello et al. 2004). Furthermore, it can also be argued that the effect of hypertension on myocardial efficiency is age‐dependent with SHR having “normal” efficiency at young age, “improved” efficiency at the adult stage, and reduced efficiency at middle‐age as compared with controls. It is not difficult to imagine that this is the case. Bing et al. (1988) showed virtually no difference in mechanical performance of the SHR, compared with Wistar and Wistar‐Kyoto, from 6‐month to 18‐month of age. However, the energetic performance of the SHR might be affected by age. Further experiments are required to test the hypotheses of “no difference in myocardial efficiency between Wistar rats and Wistar‐Kyoto rats” and “age‐dependent change of efficiency of the SHR”.

### Comparison with hypertension‐induced LV hypertrophy in human

Our results, showing reduced mechanical efficiency in aged SHR trabeculae, are not *directly* comparable to those obtained from the hearts of hypertension‐induced LV hypertrophy in patients (Ishibashi et al. 1996; Laine et al. 1999; Akinboboye et al. 2004; de las Fuentes et al. 2006). In our experiments, the quantification of mechanical efficiency of isolated trabeculae excludes the basal heat production, whereas in human studies, the efficiency unavoidably includes the basal oxygen consumption. Thus, direct comparison of in vitro tissue efficiency and in vivo whole‐heart efficiency requires the assumption that the basal metabolism is unaffected by hypertensive‐hypertrophy. In fact, Burns and Montini (1982) have shown no difference in the rate of oxygen consumption between Wistar‐Kyoto and SHR isolated ventricular myocytes. Although their results were obtained from 4‐month‐old rats, the effect of hypertensive‐hypertrophy on basal metabolism may be age‐dependent and, at the failing stage, the extent of this effect may increase. We are not aware of other studies, except Burns and Montini (1982) in young SHR, quantifying the basal metabolism in isolated SHR preparations. Hence, *direct* comparison of our results with clinical studies is limited.

Furthermore, the reason for reduced efficiency of the human hypertension‐hypertrophied heart is not unanimous. We found lower work output but no change in energy input thereby giving rise to reduced efficiency in the SHR. Akinboboye et al. (2004) reported lower work per mass, but little change in oxygen consumption, in human hypertension‐induced LV hypertrophy. We note that Witjas‐Paalberends et al. (2014) also reported lower work and no change in oxygen consumption (derived from measurements of acetate clearance) in a human hypertrophic cardiomyopathy with mutations in genes encoding myosin‐binding protein‐C and myosin heavy chain. In contrast, Laine et al. (1999), de las Fuentes et al. (2006), Ishibashi et al. (1996) reported no significant change in work output, but oxygen consumption tended to be greater in human patients. We emphasize that in human studies, measurements are necessarily constrained to a single afterload, whereas in animal studies, we have the luxury of making measurements across the complete spectrum of afterloads. This fundamental difference of experimental protocol renders consensus between human and animal results elusive.

### Conclusions

We consider it important that measurements be made across a wide range of muscle lengths or afterloads, and we suggest that this practice be adopted in future for both experimental and clinical studies. With this protocol, we find that hypertension‐induced left‐ventricular hypertrophic tissues have: (1) lower stress‐length work output, arising from both the reduced ability of muscle to produce sufficient force and to shorten sufficiently; but (2) normal metabolic energy expenditure, suggesting that crossbridge heat and Ca^2+^‐ATPase heat both remain unaffected. Hence, hypertension‐induced hypertrophic left‐ventricular tissues have lower mechanical efficiency. These results, showing mechanical but not energetic compromise, may explain the reduced myocardial efficiency reported in systemic hypertensive‐hypertrophy.

## Acknowledgments

We thank A. Petzer for animal husbandry and S. Lindsay for assistance with blood pressure measurements.

## Conflict of Interest

None declared.
